# Commuting paradox of the technopole Newtown: A case study in the Seoul Metropolitan Area

**DOI:** 10.1371/journal.pone.0306304

**Published:** 2024-07-05

**Authors:** Phengsy Dalavong, Chang Gyu Choi

**Affiliations:** Graduate School of Urban Studies, Hanyang University, Seoul, Republic of Korea; AUM: American University of the Middle East, KUWAIT

## Abstract

This study examines the relationship between different types of new town development and their impact on commuting patterns. It provides an empirical analysis of how development in Technopole Newtown and Residential Newtown influences commuting time and distance. Technopole Newtown is characterized by a blend of technological institutional clusters and residential development, while Residential Newtown primarily emphasizes residential development. Seoul Metropolitan Area was chosen as the case study, with data sourced from the Household Travel Survey. This study reveals a surprising commuting paradox in Technopole Newtown, where the plan was to blend job opportunities with residential development. The findings indicate that commuters did not benefit. Instead, they endured longer commutes in both time and distance compared to those in Residential Newtown, which is typically characterized as a bedroom community. The integration of job opportunities for the development of new towns should be considered a crucial factor in creating urban sustainability in the future.

## 1. Introduction

Recent studies on new towns have highlighted significant commuting challenges for residents, such as extensive cross-commuting and long commutes [1–3]. The ideal development of a new town aims to achieve self-sufficiency by balancing residential and job opportunities, reducing the need for long commutes [4–6]. However, the ways to build new towns to achieve this balance in urban planning remain a subject of debate. This study aims to shed light on two distinct types of new town development—Residential Newtown and Technopole Newtown—and their impacts on commuting patterns.

Residential Newtown primarily focused on housing provision and was developed in response to rapid urbanization, urban sprawl, and the mitigation of housing shortages in city centers [3, 7]. Although these developments have addressed housing needs, they have also been associated with significant commuting challenges. For example, residents often experience cross-commuting [2, 3, 8, 9] and longer commutes [1, 10], highlighting the need to increase job opportunities within a new town to mitigate these issues [11].

Technopole Newtown emphasizes increasing job opportunities and housing provision. Technopoles are generally recognized for their ability to generate employment opportunities, which is largely attributed to their emphasis on technology and innovative industry clustering.[12, 13]. They often face challenges related to commuting, which prompts workers to reside within its areas [12]. These challenges of balanced development have led to the development of Technopole Newtown, which blends the employment-focused nature of technopoles with new town development principles. However, empirical research has been limited to investigating how the impact of Technopole Newtown differs from that of Residential Newtown, in terms of commuting patterns.

This study conducts an empirical examination of the Seoul Metropolitan Area (SMA), to investigate the progression of new town development and its subsequent impact on commuting patterns. The context of Seoul is marked by swift population growth: from 2.4 million in 1960 to 10 million by the late 1980s, and reaching 26 million by 2024 in SMA [14, 15]. The 1980s witnessed the advent of first-generation developments aimed at mitigating housing shortages and the escalation of housing prices [16–20]. These early new town projects prioritized large-scale development to achieve self-sufficiency, intending to reduce lengthy commutes. However, anticipated improvements in commuting patterns for residents have not been realized [9, 16]. Similar to international studies, these first-generation new towns have often been criticized for insufficient job opportunities within their development [21]. Subsequently, the second generation of new towns witnessed a shift towards more varied development types to increase job opportunities in new towns. A notable instance is Pangyo Technopole Newtown, which emerged as a pivotal project integrating IT and research jobs with residential spaces, addressing one of the critical limitations of earlier new towns by enhancing employment opportunities. In contrast, traditional models, such as residential new towns, continued to expand, albeit in more diverse locations. Collectively, the second generation—encompassing both Technopole and Residential Newtown—represents a significant advancement in Seoul’s urban development. This generation provides a valuable comparative basis for assessing the impact of these development models on commuting patterns.

Commuting patterns in new town developments require an understanding of the characteristic bidirectional commuting flow of the inflow and outflow commuters. Inflow commuters refer to individuals who live outside a new town and commute to work there. Outflow commuters reside in new towns and commute to workplaces elsewhere. However, commuting studies have often overlooked the inflow group, and predominantly focus on outflow commuters [3, 9, 10]. Our analysis in the literature review indicates only few studies pay attention to inflow commuters [2, 12], except for one study that investigated both groups [22]. However, this study did not emphasize the context of new town development. This indicates a significant gap in comprehensive research encompassing both inflow and outflow commuter groups.

Our study, therefore, hypothesizes that if Technopole Newtown effectively integrates job opportunities with residential development, both inflow and outflow commuter groups could benefit from shorter commuting time and distance, compared to those in Residential Newtown. This study utilizes the Household Travel Survey, which provides data on respondents’ commuting patterns, including origin–destination datasets and socioeconomic backgrounds. A multiple regression model was used for the analysis.

The remainder of this paper is organized as follows. Section 2 reviews the literature on commuting and new towns in various contexts. Section 3 presents the data sources, variables, and methodology. Section 4 presents the results of the analysis, and Section 5 discusses the findings and their policy implications. Finally, Section 6 summarizes the study’s contributions, limitations, and recommendations for future research.

## 2. Literature review

### 2.1 Job-housing balance and commuting pattern

The types of new town development and their impacts on commuting have been widely studied in the urban and transportation planning fields. These developments can be categorized based on classic job-housing ratio measurements: job-rich new towns, housing-rich new towns (often referred to as bedroom communities), and job-housing-balanced new towns.

The relationship between job and housing ratio and commuting was significant, with an imbalance in both job-rich and housing-rich areas, demonstrating similar negative impacts on commuting. These impacts are characterized by increased cross-commuting and extended commuting time and distance [2, 3, 10, 23, 24]. However, the job-housing balance has led to mixed outcomes.

In principle, the goal of achieving a job-housing balance in new town development is to bring job opportunities closer to residential areas, thereby reducing daily commuting from a transportation perspective. Empirical studies on the job-housing balance suggest mixed results on commuting. On the one hand, the job-housing balance could play a pivotal role in mitigating commuting issues. A study by Cervero and Duncan [4], conducted in the San Francisco Bay Area, found that a job-housing balance reduces travel more than retail-housing mixing. Similar findings were confirmed by Dubin [25], and Horner and Mefford [26], who also confirmed the impact of job-housing balance on reducing commuting time. These observations were further substantiated by investigating the job-housing balance of Chinese cities from the perspective of the *Danwei* policy. Zhao et al. [27] highlighted a statistically significant and negative relationship between job-housing balance and commuting time, suggesting that workers living in *Danwei* housing experience shorter commuting time than those who do not.

The job-housing balance, on the other hand, is less significant in reducing commuting. Giuliano and Small [28] studied commuting patterns for the Los Angeles region in 1980. They concluded that other factors must be more important to location decisions and that policies aimed at changing the jobs-housing balance will have only a minor effect on commuting. These findings indicate fluctuations in the impact of job-housing balance on commuting. Cervero’s [22] study of the Bay Area demonstrated that despite achieving a nearly perfect job-housing balance ratio in several Bay Area cities, less than a third of workers reside locally, with an even smaller proportion of residents working locally. Peng [29] suggested that the effect of job-housing balance on Vehicle Miles Travel (VMT) can vary depending on the job-housing ratio. His analysis revealed that job-housing ratios below 1.2 or above 2.8 significantly influenced VMT, whereas a balanced ratio between 1.2 and 2.8 caused minimal change [29]. Giuliano [30] found a weak impact between job-housing balance and traffic issues, and Ewing [31] found that the job-housing balance had a minimal correlation with average commuting time when socio-demographic variables were taken into account.

### 2.2 Residential and technopole Newtown

New town development has various origins and types, including military, industrial, economic, and residential [7]. Among these, residential new towns play a significant role in urban development and were conceived to mitigate the pressures of population growth and housing shortages in large metropolitan areas [7, 32]. The concept of new towns can be reasonably traced back to the Garden City Movement proposed by Howard Ebenezer. Ebenezer’s [6] innovative idea of creating self-sufficient communities surrounded by "Green Belts," which combined the advantages of towns and countries, provided a blueprint for new town development worldwide. Historical evidence demonstrates that the new town has considerably improved living conditions by providing much-needed housing and enhancing the overall quality of living.

Achieving significant self-sufficiency and balanced development in these new towns still poses considerable challenges [1, 3, 9, 33]. New towns, particularly those with a housing focus, often suffer from a lack of job opportunities, leading to the term “Residential Newtown” or “Bedroom Community.” The dearth of job opportunities and other urban amenities in residential towns forces residents to commute to existing centers [2, 3]. Hui and Lam [3] highlighted issues in new town development: the inadequate provision of jobs and schools. This shortfall leads to widespread cross-commuting to older, more established urban regions and city centers. This situation resulted in a functional mismatch, where new towns, originally designed to be self-sufficient, became dependent on satellite towns [34]. Stockholm’s Newtown experienced a small share of workers living there, but even smaller shares of residents worked where they lived [2]. Moreover, studies in China by Li and Zhao [10] compared different types of new towns and found that residential ones tend to have longer commuting distances and time. than other types of new towns in Beijing [10].

Technopoles, however, have originated as hubs for research, innovation, transfer, and economic competitiveness with a high-technology industrial base [12, 13, 35, 36]. Their role in job creation and economic enhancement through technology and innovation is well documented [12, 13, 37]. Silicon Valley in the United States is a prime example, fostering numerous successful tech companies and driving global technological advancements. The impact of Technopoles on commuting patterns presents a significant challenge. The rapid increase in job creation within Technopoles has led to an imbalance between job opportunities and housing provision. This imbalance resulted in a sharp increase in housing costs and commuting issues [12, 22, 38]. For instance, studies in the Bay Area of the United States during 1980–1990, a region renowned for its concentration of high-tech companies, have shown that employment decentralization is not associated with shorter average commuting distance or duration [23]. Moreover, Bay Area restricts housing production, especially in fast-growing cities, which has in many instances raised housing prices, displaced workers, and increased the average commuting distance [22]. A similar pattern was observed in an international study of Technopoles. Castells and Hall [12] described technopoles as primarily centered around workplaces, leading to a trend where workers live outside the technopoles to access urban amenities. This is evident in areas such as Taedok Science Town in Korea, Sophia-Antipolis in France, and Taiwan’s Science-based Industrial Park, all of which face similar commuting pattern challenges. The primary challenge is that workers residing outside these Technopoles commute to work because of the lack of affordable housing and urban amenities within them [12]. Similar to Residential Newtown, Technopoles have created a spatial imbalance; however, in this case, there is a shortage of housing rather than jobs.

This spatial imbalance led to the development of the Technopole Newtown model, which aims to balance between new town development by integrating the employment-focused nature of Technopoles, with the residential new town development principles. This innovative approach aims to create a balanced urban ecosystem that offers residents ample opportunities to work close to their homes. Technopole Newtown represents a promising direction that aims for self-containment by balancing housing and job opportunities. This approach can potentially reduce long commutes. Despite its expected significance, this topic remains to be investigated. Thus, this study aims to address this gap in the urban planning literature.

### 2.3 New town development in Seoul Metropolitan Area

Since the 1960s, following the Korean War, the SMA has experienced rapid industrialization and urbanization, leading to significant housing shortages and skyrocketing housing prices [17–20]. In response to the acute need for housing and to stimulate economic growth, 15 massive new towns were developed as second-generation new towns in the SMA, collectively comprising approximately three million people [17, 20]. Moreover, a third-generation new town is currently being planned. These developments aim to meet housing demands, stabilize housing prices, curb urban sprawl, and archive self-containment, reflecting the evolution of the new town development model over time.

The first generation of new towns, initiated in the 1980s, comprised five developments that were significantly influenced by greenbelt regulations [9, 16]. This influence necessitated the location of the new towns beyond the greenbelt, leading to a "Leap-frog" development pattern, characterized by disconnection from Seoul’s continuous urban fabric [16]. The primary goal of these towns is to achieve self-containment, emphasizing the large scale of new towns to reduce commuting. Creating self-contained new towns has proven advantageous in minimizing non-commuting travel, as studies indicate that residents within these five towns largely conduct their travel activities locally [39]. However, in terms of commuting, larger new town developments do not offer more benefits than mid-sized new towns, especially for travel to Seoul [9]. Moreover, long commutes in first-generation new towns have been identified as a critical issue [16].

During the 2000s, South Korea embarked on the development of second-generation new towns, which marked a significant shift towards a wider variety of new towns with a focus on sustainability [20, 40]. Unlike their first-generation counterparts, these new towns combined mixed housing and increased job opportunities within them, while also capitalizing on their geographic advantages to create thriving regional hubs. Pangyo Newtown, for example, adopted a mixed-use approach that harmonized residential spaces with commercial and IT industries, embodying Korea’s Techno Valley [41]. Despite these innovations, second-generation new towns still aimed for self-sufficiency and a balance between jobs and housing [20], but with a greater emphasis on diverse locations. For instance, second-generation new towns had two main locations: those close to the old urban core, such as Wirye and Pangyo new towns, and those further away. Wirye Newtown, located near the urban core, focused on residential expansion and blending urban accessibility with residential needs. Meanwhile, future new towns aimed to reduce Seoul’s dominance by promoting the development of self-contained regional hubs, as evidenced by the emergence of Dongtan1, Dongtan2, and Okjeong/Hoecheon [20]. Additionally, Gimpo Newtown and Paju Newtown were designed to be integrated with existing areas to achieve balanced development across the Han River [20, 40]. Although second-generation new towns exhibit greater diversity than first-generation, their influence on commuting patterns has not been researched.

Our study addresses this gap by focusing on three new towns under second-generation new town development—Pangyo, Wirye, and Gimpo—to delineate the “Technopole Newtown” and “Residential Newtown” models. Pangyo Newtown, integrating the residential, commercial, and IT sectors, is a prime example of the “Technopole Newtown.” It attracted over 600 IT companies and created 43,000 jobs, demonstrating a balanced blend of residential and job opportunities, especially by integrating Regional Innovation Systems managed by Gyeonggi province’s local government [20, 42–44]. Conversely, Wirye and Gimpo’s new towns exemplify the "Residential Newtown" model, focusing primarily on residential development. Wirye, strategically positioned between Seoul and Gyeonggi Province, aimed to alleviate housing shortages near the Kangnam Business District (KBD), approximately 10.7 km away, and accommodated approximately 110,000 residents within a 6.8 km^2^ area [20, 45]. Gimpo Newtown, located about 20 km from the Yeouido Business District (YBD), focused on rejuvenating the area to address housing shortages, housing approximately 145,480 residents in an area of approximately 11.7 km^2^ with a mix of low- and high-rise apartments [20, 40] (Table 1).

**Table 1:** Characteristics of new town development.

Characteristics	Pangyo	Wirye	Gimpo
Representative types of new town	Technopole Newtown	Residential Newtown	Residential Newtown
Development period	2003–2017	2008–2020	2006–2017
Area (km^2^)	8.9	6.8	11.7
Population (person)	87,795	110,000	145,480
Distance to nearest business centers (CBD, KBD, YBD)	13km	10.7km	24km

Source: Ministry of Land, Infrastructure and Transport (MOLIT) [20]

## 3. Data source, variables, and methodology

### 3.1 Commuter groups setting

**Fig 1:**
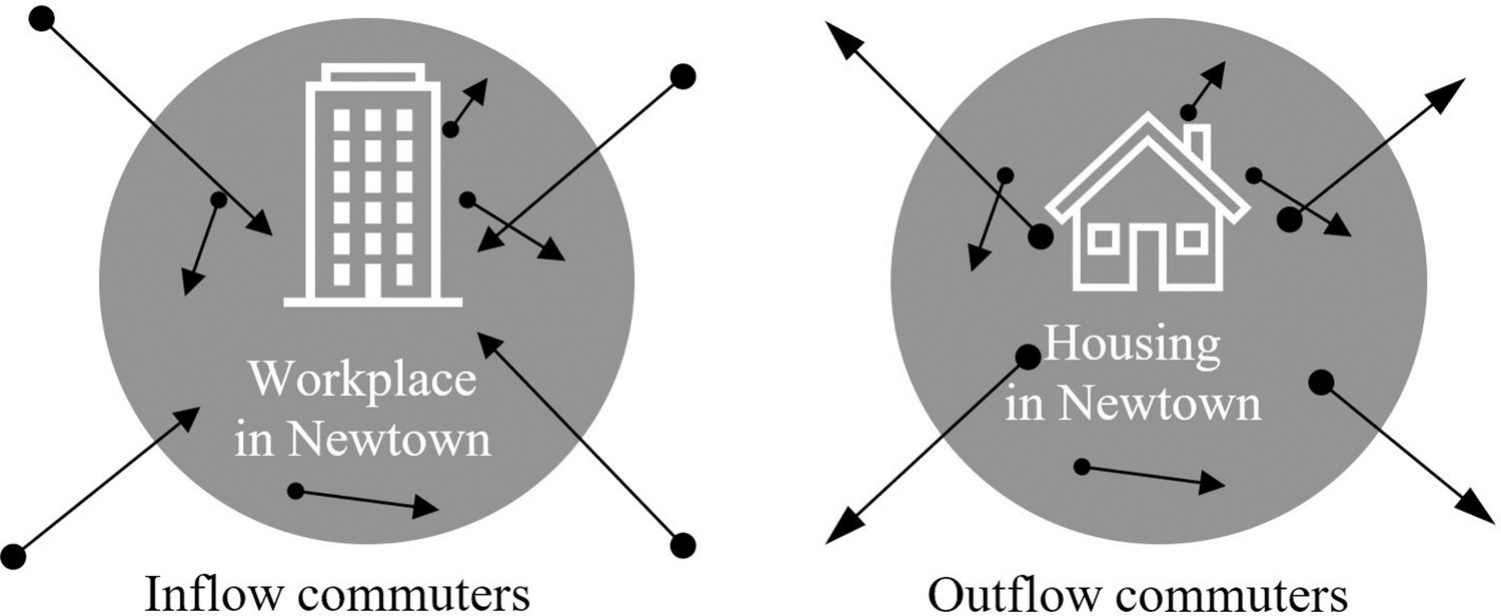
The characteristics of inflow and outflow commuters.

Effective planning for new towns and transportation planning requires an understanding of the bidirectional pattern of commuters—Inflow and Outflow—commuters. Inflow commuters are those who travel to a new town to work in another location. Outflow commuters refer to residents of a new town who commute to work elsewhere (Fig 1). These patterns not only reflect commuting behaviors, but also highlight the interactions between residential and work locations. A high rate of outflow commuting suggests that residents of a new town frequently travel to other areas to search for suitable job opportunities or other urban amenities. Such patterns of outflow commuting are often observed in housing-rich areas worldwide, as noted in previous studies [2, 3, 10]. In contrast, a high rate of inflow commuting indicates that the new town is successfully attracting a workforce from the surrounding areas, has high job opportunities, and is especially notable in job-rich areas.

Mainstream research has predominantly focused on outflow commuting from new towns [3, 9, 10, 46, 47], with only a few studies examining inflow commuting [12], or both inflow and outflow commuting groups [2]. This indicates a significant gap in the comprehensive research that encompasses both groups and underscores the importance of this study.

### 3.2 Data and methodology

This study used the 2016 Household Travel Survey datasets from the Korea Transportation DataBase (KTDB), which compiles basic data to conduct travel demand analysis on a nationwide scale every five years. This study adhered to the general principles of ethical research that are appropriate for studies classified as minimal risk. Our study did not access identifiable personal information from the dataset, thereby posing minimal or no risk to the participants.

We selected weekday data for commuting from home to the workplace during one-way trips. Commuting time was the actual travel time of the respondents’ answers, from the starting point at home to the destination at the workplace, for all modes of transportation. Additionally, we calculated the distance between home and workplace using Euclidean distance, with the centroid of the home’s administrative boundary “*Dong*” as the origin and of the workplace’s administrative boundary as the destination. *Dong* refers to the smallest administrative boundary in South Korea. We limited the O-D of commuting within the SMA.

This study prioritized the logarithms of commuting time and distance as the dependent variables to address the normal distribution of commuting time and distance. To control for factors that could influence commuting patterns, this study reviewed previous research and included socioeconomic characteristics, public transportation accessibility, and mode choice [9, 48–50]. Accessibility was assessed by examining the access time to bus stops, and transportation mode choice was based on the type of transportation used by commuters, thus offering insights into public transportation planning in the region [49]. Socioeconomic characteristic variables included age, gender, income (unit: million Korean Won per month), household members, and jobs [9, 10, 51]. Housing type and vehicle ownership were identified as indicators of commuter property status.

The analysis employed a multiple regression model to investigate how different types of new towns impact commuting time and distance while controlling for socioeconomic factors. Equations were then separately estimated for different commuter groups, to uncover any differences between Technopoles and Residential Newtown. Furthermore, the analysis examined how personal characteristics relate to commuting patterns, and provided additional tests for the household responsibility hypothesis. The multiple regression model equation is as follows:

$$log({\;Y}_{t})={\;\beta }_{0}+{\;\beta }_{1}{\;X}_{1}+{\;\beta }_{2}{\;X}_{2}+\ldots +{\;\beta }_{10}{\;X}_{10}+\epsilon$$ (1)

$$log({\;Y}_{d})={\;\beta }_{0}+{\;\beta }_{1}{\;X}_{1}+{\;\beta }_{2}{\;X}_{2}+\ldots +{\;\beta }_{10}{\;X}_{10}+\epsilon$$ (2)

$$log({\;Y}_{t^{\prime }})={\;\beta }_{0}+{\;\beta }_{1}{\;X}_{1}+{\;\beta }_{2}{\;X}_{2}+\ldots +{\;\beta }_{10}{\;X}_{10}+\epsilon$$ (3)

$$log({\;Y}_{d^{\prime }})={\;\beta }_{0}+{\;\beta }_{1}{\;X}_{1}+{\;\beta }_{2}{\;X}_{2}+\ldots +{\;\beta }_{10}{\;X}_{10}+\epsilon$$ (4)

In this model, Y_t_ and Y_d_ represent the logarithms of commuting time and distance for inflow commuters, respectively. Y_t′_ and Y_d′_ denote the same logarithms for outflow commuters. X1 is the main independent variable of interest, which is the new town development type. X2 represents the transportation mode choices. X3 refers to the accessibility to public transit as access time to bus stops, for which we use the logarithmic form to create a normal distribution of data, and X4 to X10 are the socioeconomic characteristics of the commuters used as control variables. β_1_ to β_10_ represent the coefficients of each explanatory variable, and ϵ is the error term of the model.

## 4. Results

### 4.1 Descriptive analysis

The validation sample includes 937 inflow commuters and 987 outflow commuters. Among the inflow commuters, 65.2% commute to Technopole Newtown, while the remainder are distributed between Wirye (17.9%) and Gimpo (16.9%). Conversely, the outflow of commuters predominantly originates from Gimpo Newtown (46%), followed by Wirye Newtown (29.5%), and Technopole Newtown (24.5%). Although a larger percentage of inflow commuters enter Technopole Newtown compared to Residential Newtown, it experiences a smaller outflow of people. This distinction emphasizes the significance of examining both the inflow and outflow of commuters to comprehensively assess commuting patterns and promote balanced development in new towns.

**Fig 2:**
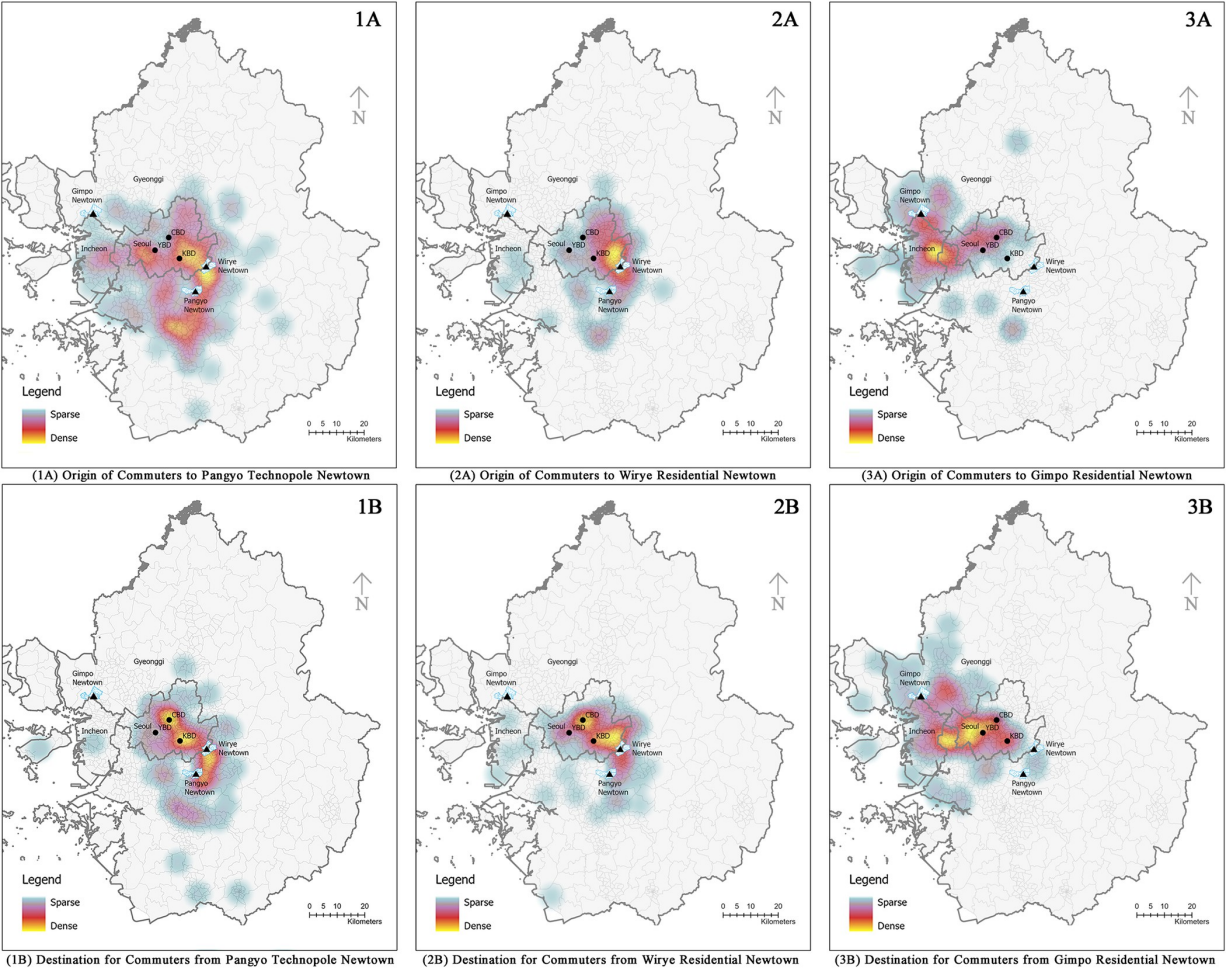
Heatmap of origins and destinations of inflow and outflow commuters. The heatmap indicates the origins of inflow commuters heading to Pangyo Technopole (1A), Wirye Residential Newtown (2B), and Gimpo Residential Newtown (3C). Conversely, it shows the destinations of outflow commuters coming from Pangyo Technopole Newtown (1B), Wirye Residential Newtown (2B), and Gimpo Residential Newtown (3B). The map was created by manually using QGIS.

Where they commute from and to is crucial for understanding the influence of their location on commuting behaviors between different new towns. For example, the distribution of commuter locations for those commuting to Technopole Newtown is widely dispersed across the SMA, as shown in Fig 2 (1A). Residential Newtown experiences more concentrated population inflows from the Kangnam area for Wirye Residential Newtown, and the city of Incheon for Gimpo Residential Newtown, as illustrated in Fig 2 (2A) and Fig 2 (3A), respectively. On the other hand, the majority of outflow commuters from these three new towns tend to move towards the central business district of Seoul. In particular, those from Pangyo Technopole Newtown primarily worked in Seongnam, the KBD, and the CBD, as shown in Fig 2 (1B). Residents of Wirye Residential Newtown typically travel to the KBD and CBD areas (Fig 2 (2B)), while those from Gimpo Residential Newtown usually commute to the city of Incheon and YBD (Fig 2 (3B)). The residential and work locations of commuters’ exhibit a cross-commuting phenomenon, which reflects the interplay between inflow and outflow patterns. This can be observed in the impact of new town development on different types of commuters.

**Fig 3:**
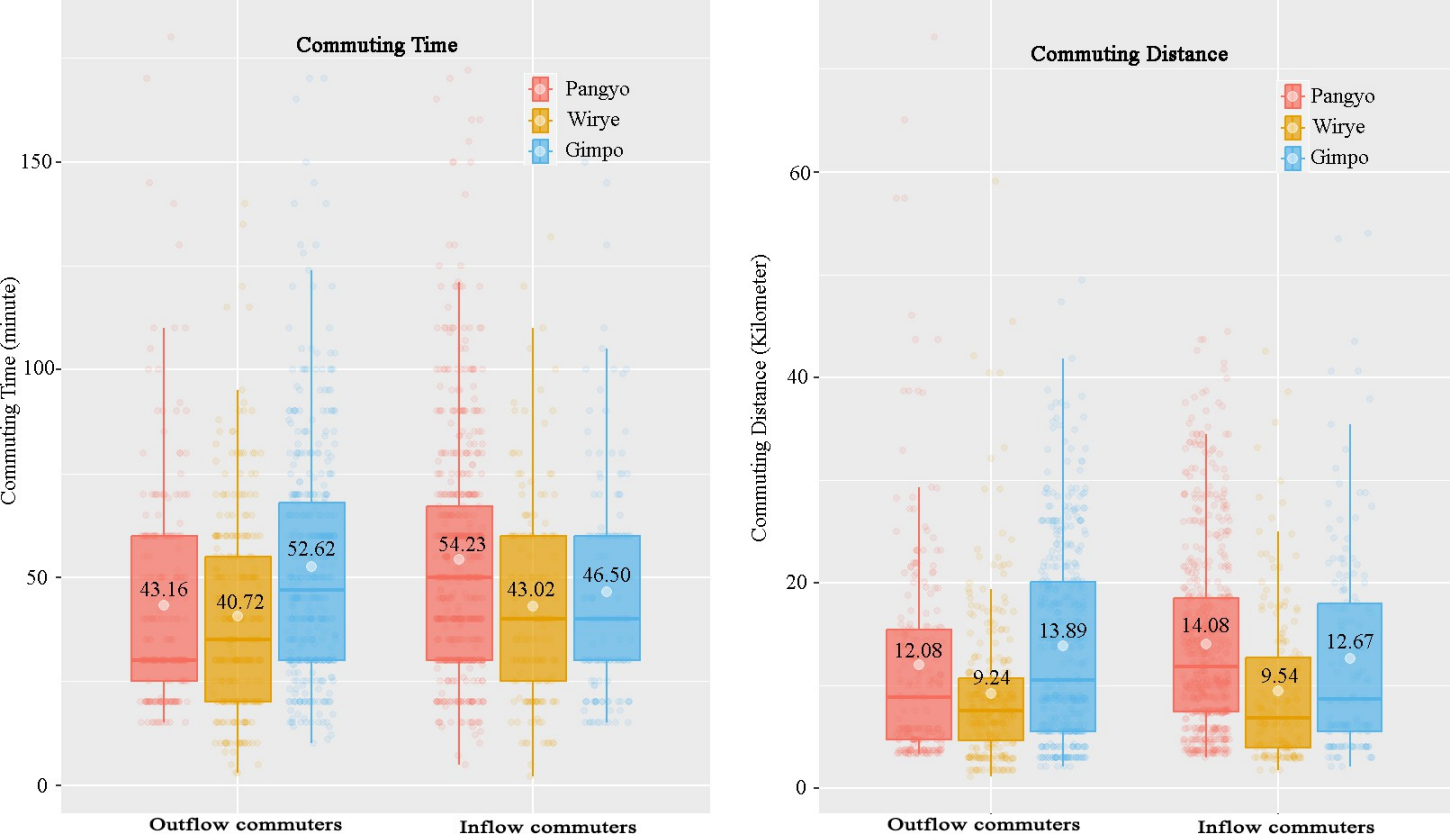
Commuting time and distance of inflow and outflow commuters by new towns. Differences are all statistically significant. *Left*: boxplots of commuting time. *Right*: boxplots of commuting distance.

Additionally, notable differences are observed in commuting time and distance. Those commuting to Technopole Newtown experience longer commutes in terms of both time and distance, than those commuting to Residential Newtown. The average commute time and distance are as follows: Pangyo (54.23 minutes; 14.08 km), Gimpo (46.50 minutes; 12.67 km), and Wirye (43.02 minutes; 9.54 km). Outflow commuters from Gimpo Newtown have longer commutes than those from Technopole Newtown and Wirye Newtown, averaging 52.62 minutes/13.89 km, 43.16 minutes/12.08 km, and 40.72 minutes/9.24 km, respectively (Fig 3). These differences in commuting are statistically significant between the development types. They present significant challenges for the development of Technopole Newtown from a commuting perspective, aiming to reduce commuting time and distance as a guiding principle.

From a transportation perspective, commuters typically take an average of 4–6 minutes to access a bus stop. However, the mode of transportation demonstrates a preference for private cars for both inflow and outflow commuters in each new town. For instance, 65% of inflow commuters to Gimpo Residential Newtown use private cars followed by 55% in Technopole Newtown, and 40% in Wirye Residential Newtown (Table 2). This trend is even more pronounced among outflow commuters, with more than half of them utilizing private cars in these new town developments (58%, 51%, and 56%, respectively). Vehicle ownership is over 80% in all groups, reflecting a reliance on private vehicles. This may be attributed to the fact that new towns are still in the process of being developed. Nevertheless, using public transportation for commuting to and from these areas is crucial to rising commuting needs. It could serve as a significant alternative method to contribute towards the environmental sustainability of new town development, especially when self-containment proves challenging to achieve.

**Table 2:** Commuting profiles of inflow and outflow commuters.

	Inflow commuters (n = 937)				Outflow commuters (n = 987)			
Variables	Pangyo (n = 611)	Wirye (n = 168)	Gimpo (n = 158)	F-test/ chi-square	Pangyo (n = 239)	Wirye (n = 291)	Gimpo (n = 457)	F-test/ chi-square
	Mean (SD)/ n (%)	Mean (SD)/ n (%)	Mean (SD)/ n (%)		Mean (SD)/ n (%)	Mean (SD)/ n (%)	Mean (SD)/ n (%)	
Commuting time (minute)	54.2 (27.9)	43.0 (24.0)	46.5 (26.2)	F = 13.9***	43.2 (26.8)	40.7 (23.6)	52.6 (27.5)	F = 21.3***
Commuting distance (km)	14.1 (9.0)	9.5 (7.8)	12.7 (10.1)	F = 17.0***	12.1 (11.0)	9.2 (7.4)	13.9 (9.4)	F = 22.2***
Mode of transportation				x^2^ = 38.9***				x^2^ = 7.0
Private car	333 (55%)	67 (40%)	103 (65%)		122 (51%)	163 (56%)	265 (58%)	
Public transit	231 (38%)	72 (43%)	44 (28%)		97 (41%)	106 (36%)	169 (37%)	
Walking and cycling	44 (7.2%)	26 (15%)	6 (3.8%)		18 (7.5%)	20 (6.9%)	18 (3.9%)	
Others	3 (0.5%)	3 (1.8%)	5 (3.2%)		2 (0.8%)	2 (0.7%)	5 (1.1%)	
Access time to bus stop (minute)	5.2 (2.6)	5.5 (2.8)	5.6 (3.4)	F = 1.5	4.6 (2.1)	5.4 (2.7)	4.9 (2.4)	F = 7.8***
Age	40.2 (10.0)	44.9 (11.4)	48.8 (10.8)	F = 48.9***	44.1 (10.2)	43.2 (11.1)	44.0 (11.0)	F = 0.6
Gender				x^2^ = 4.3				x^2^ = 2.4
Female	162 (27%)	48 (29%)	55 (35%)		82 (34%)	91 (31%)	131 (29%)	
Male	449 (73%)	120 (71%)	103 (65%)		157 (66%)	200 (69%)	326 (71%)	
Income				x^2^ = 18.4**				x^2^ = 21.4***
Less than 3 million	161 (26%)	53 (32%)	66 (42%)		54 (23%)	85 (29%)	93 (20%)	
3–5 million	206 (34%)	63 (38%)	49 (31%)		82 (34%)	108 (37%)	220 (48%)	
More than 5million	244 (40%)	52 (31%)	43 (27%)		103 (43%)	98 (34%)	144 (32%)	
Job				x^2^ = 181.5***				x^2^ = 91.9***
Office worker	426 (70%)	70 (42%)	44 (28%)		125 (52%)	134 (46%)	218 (48%)	
Specialized skill	75 (12%)	16 (9.5%)	7 (4.4%)		38 (16%)	12 (4.1%)	12 (2.6%)	
Service worker	31 (5.1%)	24 (14%)	35 (22%)		25 (10%)	37 (13%)	66 (14%)	
Salesman	41 (6.7%)	20 (12%)	33 (21%)		26 (11%)	80 (27%)	83 (18%)	
Manual worker	30 (4.9%)	36 (21%)	33 (21%)		13 (5.4%)	26 (8.9%)	66 (14%)	
Others	8 (1.3%)	2 (1.2%)	6 (3.8%)		12 (5.0%)	2 (0.7%)	12 (2.6%)	
Household members	3.1 (1.1)	3.0 (1.0)	3.0 (1.1)	F = 0.1	2.9 (1.0)	2.8 (1.1)	3.3 (1.0)	F = 28.7***
Housing type				x^2^ = 50.9***				
Apartment	423 (69%)	78 (46%)	104 (66%)		227 (95%)	191 (66%)	436 (95%)	x^2^ = 185.0***
Multi-family housing	50 (8.2%)	23 (14%)	14 (8.9%)		1 (0.4%)	25 (8.6%)	3 (0.7%)	
Single-family housing	58 (9.5%)	44 (26%)	20 (13%)		4 (1.7%)	55 (19%)	16 (3.5%)	
Row house | Villa	59 (9.7%)	20 (12%)	20 (13%)		2 (0.8%)	20 (6.9%)	2 (0.4%)	
Others	21 (3.4%)	3 (1.8%)	0 (0%)		5 (2.1%)	0 (0%)	0 (0%)	
Vehicle ownership				x^2^ = 24.7***				x^2^ = 6.2**
No	71 (12%)	45 (27%)	30 (19%)		20 (8.4%)	44 (15%)	50 (11%)	
Yes	540 (88%)	123 (73%)	128 (81%)		219 (92%)	247 (85%)	407 (89%)	

Notes

*p<0.1

**p<0.05

***p<0.001

Socioeconomic characteristics reveal notable age differences between the inflow and outflow commuter groups, with the inflow group tending to be younger, particularly in Technopole Newtown. This younger demographic typifies the workforce commuting to new towns, particularly Technopole Newtown, which attracts younger talent to jobs in the IT, innovation, and R&D sectors. The Pangyo Technopole Newtown data reveal that those who have earned more than 5 million won comprise a higher percentage of the population compared to residents in other new towns. For instance, 40% of inflow commuters earning more than 5 million won commute to the Pangyo Technopole Newtown, while those earning less than 3 million won commute to the Gimpo Residential Newtown. Conversely, the income pattern of outflow commuters for Pangyo and Wirye Resident Newtowns is similar to that of inflow commuters, while Gimpo Newtown show a slight concentration of those earning 3–5 million won (48%), and exhibit statistical significance between these groups. This income discrepancy may be correlated with their occupations. Technopole Newtown predominantly hosts office workers, mainly employed in technology and research companies, while service jobs, salesmen, and manual workers are more common in Residential Newtown. In short, Pangyo Technopole Newtown embodies the ideal of a modern new town development that emphasizes information technology, innovation, and research-oriented employment opportunities. These types of jobs often offer higher salaries and cater to a younger demographic, as opposed to the traditional new towns that primarily concentrate on providing housing and accommodating commuters who work in other locations.

### 4.2 Multiple regression model

An analysis using multiple regression models was conducted to explore the impact of Technopole and Residential Newtown on their respective inflow and outflow commuting patterns. This comprehensive analysis, which included two distinct models for inflow and outflow commuters, revealed surprising commuting paradox trends within Technopole Newtown.

Regrading inflow commuters, our analysis results were contrary to the anticipated benefits of Technopole Newtown’s integrated job-residential planning when taking socio-economic factors into account. The explanatory model for commuting time accounted for 28% (adjusted R^2^ = 0.280) of the variance, and that for commuting distance accounted for 16% (adjusted R^2^ = 0.160). Both the commuting time and distance models were statistically significant. The outcomes for inflow commuters’ travel time and distance revealed that Technopole Newtown experiences longer commutes than Residential Newtown. For instance, compared to Wirye Residential Newtown, commuters to Technopole Newtown experienced significantly longer commuting time and distance, with coefficients of -0.260 and -0.367, respectively. Moreover, in comparison with Technopole Newtown, Gimpo Residential Newtown demonstrated shorter commuting time and distance, with coefficients of -0.094 and -0.159, respectively (Table 3). A similar pattern was observed in the San Francisco Bay Area, which had experienced rapid growth in suburban employment. Much of this growth was concentrated in sub-centers characterized by high concentrations of research, innovation, and IT companies, leading to longer commutes for workers [23]. These outcomes underscore a commuting paradox in Technopole Newtown, where the integration of residential and job opportunities, has not translated into shorter commutes.

**Table 3:** Multiple regression of inflow commuters.

Variables	Commuting time (log)		Commuting distance (log)	
* *	Coef.	std. Error	Coef.	std. Error
(Intercept)	3.626 ***	0.120	2.538 ***	0.164
Type of new towns: Pangyo Technopole Newtown	-	-	-	-
Wirye Residential Newtown	-0.260 ***	0.045	-0.367 ***	0.061
Gimpo Residential Newtown	-0.094 *	0.048	-0.159 **	0.066
Mode of transportation: Private car	-	-	-	-
Public transit	0.540 ***	0.037	-0.168 ***	0.050
Walking and cycling	-0.009	0.063	-0.570 ***	0.085
Others	-0.046	0.150	0.165	0.204
Access time to bus stop (log)	0.019	0.039	-0.021	0.053
Age	-0.003 *	0.002	-0.006 ***	0.002
Gender: Female	-	-	-	-
Male	0.112 ***	0.037	0.202 ***	0.050
Income: less than 3 million	-	-	-	-
3–5 million	0.038	0.042	0.085	0.058
more than 5 million	0.027	0.044	0.033	0.060
Job: Office Worker	-	-	-	-
Specialized skill	-0.076	0.053	-0.111	0.072
Service worker	-0.054	0.058	-0.062	0.079
Salesman	-0.06	0.058	-0.041	0.079
Manual worker	0.005	0.059	0.028	0.081
Others	-0.12	0.122	-0.117	0.166
Household member	0.038 **	0.017	0.071 ***	0.023
Housing types: Apartment	-	-	-	-
Multi-family housing	-0.045	0.056	-0.039	0.077
Single-family housing	-0.140 **	0.049	-0.156 **	0.067
Row house | Villa	-0.046	0.053	0.053	0.072
Others	-0.094	0.103	-0.292 **	0.140
Vehicle ownership: No	-	-	-	-
Yes	-0.058	0.052	-0.092	0.07
Observations	937		937	
R^2^ / R^2^ adjusted	0.296 / 0.280		0.179 / 0.161	
log-Likelihood	-622.508		-911.554	

* p<0.1

** p<0.05

*** p<0.01

The commuting mode choices of inflow commuters significantly affected their commuting patterns. The results indicate that inflow commuters using public transit tended to travel shorter distances, yet the commute time was longer compared with those using private cars. Commuters seem to have preferred private cars for their speed, aiming to reduce commuting time; however, the rationale behind this finding has not been explored in this study. Alternatively, new towns may not have sufficiently developed to offer the level of comfort needed to meet inflow commuters’ needs, making private car use more attractive (Table 2). Interestingly, the time taken to access a bus stop did not significantly affect the commuting patterns of inflow commuters.

Meanwhile, regarding outflow commuters, the explanatory model for outflow commuters’ commuting time accounted for 28.9% of the variance (adjusted R^2^ = 0.289), and for commuting distance, it accounted for 16.5% of the variance (adjusted R^2^ = 0.165). Both models were statistically significant. The results of the analysis of outflow commuters highlight the diversity of commuting experiences between Technopole Newtown and Residential Newtown. Technopole Newtown residents experienced a longer commuting distance than Wirye Residential Newtown residents (coefficient: -0.133), although the differences in commuting time were not statistically significant. However, Technopole Newtown indicated commuting benefits for outflow commuters, compared to Gimpo Residential Newtown, as the results suggested that Technopole Newtown had shorter commuting time and distances than Gimpo Residential Newtown (coefficients: 0.252 and 0.218, respectively), as detailed in Table 4. These mixed outcomes for outflow commuters appear to favor Technopole Newtown development over inflow commuters, although the benefits remain unclear. This observation highlights a limitation regarding Technopole Newtown’s suggestion that the integrated job-residential model, aimed at balanced development to enhance commuting efficiency, is not supported by our hypothesis.

**Table 4:** Multiple regression of outflow commuters.

Variables	Commuting time (log)		Commuting distance (log)	
* *	Coef.	std. Error	Coef.	std. Error
(Intercept)	3.231 ***	0.125	1.800 ***	0.178
Type of new towns: Pangyo Technopole Newtown	-	-	-	-
Wirye Residential Newtown	-0.048	0.047	-0.133 **	0.067
Gimpo Residential Newtown	0.252 ***	0.042	0.218 ***	0.060
Mode of transportation: Private car	-	-	-	-
Public transit	0.558 ***	0.037	-0.052	0.053
Walk and cycling	0.096	0.071	-0.412 ***	0.101
Others	-0.280 *	0.169	-0.585 **	0.241
Access time to bus stop (log)	0.041	0.037	0.056	0.053
Age	-0.001	0.002	-0.003	0.002
Gender: Female	-	-	-	-
Male	0.091 **	0.037	0.125 **	0.052
Income: Less than 3 million	-	-	-	-
3–5 million	0.085	0.047	0.137 **	0.066
more than 5 million	0.168 ***	0.050	0.254 ***	0.072
Job: Office Worker	-	-	-	-
Specialized skill	0.106	0.068	0.193 **	0.098
Service worker	-0.194 ***	0.051	-0.165 **	0.073
Salesman	-0.167 ***	0.044	-0.267 ***	0.063
Manual worker	-0.144 **	0.058	-0.332 ***	0.083
Others	0.069	0.101	-0.028	0.145
Household members	0.010	0.018	0.071 ***	0.025
Housing types: Apartment	-	-	-	-
Multi-family housing	0.209 **	0.100	0.116	0.142
Single-family housing	0.047	0.064	0.005	0.091
Row house | Villa	0.171	0.106	0.069	0.152
Others	-0.199	0.227	-0.292	0.323
Vehicle ownership: No	-	-	-	-
Yes	-0.054	0.057	0.061	0.082
Observations	987		987	
R^2^ / R^2^ adjusted	0.304 / 0.289		0.183 / 0.165	
log-Likelihood	-682.697		-1033.443	

* p<0.1

** p<0.05

*** p<0.01

The analysis of transportation modes for outflow commuters revealed a consistent pattern with inflow commuters. Individuals using public transport tend to travel shorter distances but face longer commuting time than those using private vehicles. This similarity in commuting experiences between the inflow and outflow groups could be attributed to the effects of new town development, where both groups encountered challenges with public transportation during the first or last mile of their journeys in new towns.

The socioeconomic characteristics of outflow commuters indicate that residents of new towns, with incomes exceeding 5 million, have longer commuting time and distance. These findings reflect the commuting patterns in the SMA, demonstrating that individuals commute further to the city center, where they encounter more job opportunities and have the potential for higher earnings. This trend is consistent across different job sectors, with those employed in the service sector and manual labor having shorter commutes than office workers.

## 5. Discussion

In response to persistent reports of long commutes, there is a strong initiative to develop new towns designed for a balanced mix of residential and job opportunities. This study compares the effects on commuting in such balanced new towns, focusing on Technopole Newtown, which emphasizes a blend of job opportunities in technological institutions and residential development, with Residential Newtown, which is known for its focus on housing provision. By comparing these towns, this study explores their impact on both inflow and outflow commuters.

The initial hypothesis of our study theorized that Technopole Newtown’s integrated job-residential model would lead to a decrease in commuting time and distance. Contrary to this, our findings do not support a significant benefit for commuting. For instance, outflow commuters and the anticipated benefits of the integrated job-residential model in reducing commuting time were not evident. These commuters of Technopole Newtown experience commuting time similar to those of outflow commuters from Residential Newtown (Wirye) (Table 3). Additionally, inflow commuters to Technopole Newtown endure even longer commuting time and distance than those commuting to Residential Newtown (Table 4), challenging the effectiveness of the integrated model.

One uncertainty persists following the analysis. Although the findings confirm the ambiguous benefits of Technopole Newtown, the mechanisms through which it influences the commuting patterns of inflow and outflow commuters remain unexplored. First, although an increase in job opportunities is generally positive, a mismatch between these opportunities and residents’ job preferences may hinder efforts to alleviate commuting challenges. For instance, in Technopole Newtown, the specific demand for younger people with specialized technical skills and its request to attract talent from distant areas resulted in longer commutes compared to residential towns (Table 2). These characteristics can be observed in Technopole, which is specific to single jobs prominent in which the diversity of jobs and activities also impacts the areas that lack essential urban amenities. This could lead to workers being willing to live outside the area, as they stay in the new town. This was also observed in excellent studies by Castells and Hall [12].

Second, when individuals seek employment in areas that offer a balance between housing availability and job opportunities, they often face high housing costs and lack affordable options. As in the Technopole, the Newtown location is 14 km away from the business center area, with well-connected transportation, and the development focused on prime residents’ housing developments, which could lead to extremely high housing prices in the area. This scenario forces workers to reside further away from these areas [12, 23, 33, 52], as observed in the rapidly urbanizing San Francisco Bay Area in 1980–1990. However, it does not emphasize new town development, showing a near-perfect balance in several cities and fewer workers living locally, with even fewer residents working locally [22]. Limited housing production in fast-growing cities has led to higher housing prices, displaced workers, and extended average commuting distances [22].

These results do not support the notion that “Residential Newtown” or “Bedroom Communities” were effective; rather, they suggest that the attempt by Technopole Newtown to reduce commuting has not been achieved to a large extent. Addressing these challenges requires a holistic urban planning approach that integrates job opportunities and housing requirements. For example, prioritizing diverse job opportunities and housing options within new towns could attract individuals who prefer these kinds of jobs, supporting them in staying in the area, and thereby alleviating long commutes. This approach might help balance the spatial mismatch between housing and workplaces, emphasizing co-location, and promoting synergy between work and living spaces.

## 6. Conclusions

The development of new towns to achieve a balance between jobs and residential areas, and their impact on commuting patterns, has become a critical concern for urban planners and scholars. This study contributes to this discussion by examining the types of development—Technopole Newtown and Residential Newtown—on their impact on commuting patterns, using the SMA as a case study. Technopole Newtown, which blends residential and job opportunities, aims to address the common challenges faced by new towns by offering job opportunities along with housing. This study examines its impact on commuting by comparing its patterns with those of Residential Newtown and categorizing commuters into inflow and outflow groups.

Our findings reveal distinct commuting patterns in Technopole Newtown, compared to Residential Newtown. The expected benefits of Technopole Newtown for outflow commuters are not evident, while inflow commuters experience longer commuting distances and time than those in Residential Newtown. This suggests that despite its focus on increasing job opportunities and residential development to achieve balance, Technopole Newtown’s integrated model may not significantly reduce commuting burdens for all groups. The effectiveness of Technopole Newtown from a commuting perspective is called into question, leading to a commuting paradox.

However, limitations such as the recency of the data and the lack of analysis of return trips, leisure travel, and housing prices warrant further research. Future studies that adopt more sophisticated methods can provide a more comprehensive understanding of the impact of Technopole Newtown on commuting and inform future urban planning strategies.

## Supporting information

S1 Dataset (XLSX)
